# Efficacy and Safety Aspects of Remifentanil Sedation for Intubation in Neonates: A Retrospective Study

**DOI:** 10.3389/fped.2019.00450

**Published:** 2019-11-07

**Authors:** Clément Chollat, Arielle Maroni, Marie-Stéphanie Aubelle, Cyril Guillier, Juliana Patkai, Elodie Zana-Taïeb, Aurélie Keslick, Héloïse Torchin, Pierre-Henri Jarreau

**Affiliations:** Neonatal Intensive Care Unit of Port-Royal, Paris Centre University Hospitals, APHP, Paris Descartes University, Paris, France

**Keywords:** acute pain, intubation, newborn, remifentanil, retrospective study

## Abstract

**Objective:** To evaluate the efficacy and safety of remifentanil as a premedication in neonates undergoing elective intubation.

**Study Design:** This retrospective study focused on neonates admitted to the Neonatal Intensive Care Unit of Port-Royal, Paris Centre University Hospitals, France, between June 2016 and November 2017, who received remifentanil before an elective intubation. First, atropine (10 μg/kg) was administered intravenously as a bolus, followed by remifentanil, which was administrated continuously. The dose of remifentanil was reduced twice during the study period in order to administer the minimum effective dose and thus reduce possible adverse events.

**Results:** Fifty-four neonates were exposed to remifentanil and atropine. The intubating conditions were excellent or good for 46 procedures (85%) and the median Acute Pain in Newborn Infants score was 2 (IQ 25-75: 0–5) before the sedation, 1 (0–2) during the laryngoscopy, and 0 (0–0) after the intubation. The intubation was successful at the first attempt for 18 patients (33%). Chest wall rigidity occurred in 6 procedures (11%), other respiratory problems in 5 (9%), and laryngospasm in 1 (2%). Some of the procedures were complicated by bradycardia (23%) or desaturation (37%).

**Conclusions:** Remifentanil and atropine prior to intubation provided satisfactory intubating conditions in neonates. Nevertheless, severe adverse effects (such as chest wall rigidity) are a potential risk, possibly related to the total dose received. These data do not support the safety of using remifentanil alone prior to intubation in neonates.

## Introduction

Intubation is a painful and stressful procedure that is performed daily in neonatal intensive care units (NICUs) when preterm or full-term neonates require mechanical ventilation. This invasive procedure is associated with a number of physiological side effects including hypoxemia, bradycardia, laryngospasm, bronchospasm, apnea, systemic, and pulmonary and intracranial hypertension (1). Premedication before elective intubation is recommended by the American Academy of Pediatrics to reduce pain and side effects associated with the procedure and to facilitate intubation, but there is no consensus regarding the specific pharmacological entity or the cocktail of medications that should be used (2). The specific premedication rate before neonatal intubation was only 56% in the French observational study EPIPPAIN. and mostly included opioids (67%) and midazolam (53%) (3). The ideal premedication should have a rapid onset, strong analgesic potency, and no short- or long-term adverse effects.

Remifentanil, a potent selective μ-opioid receptor agonist, is potentially a good candidate for premedication before neonatal intubation. Remifentanil has a rapid onset of action (1–2 min), a short half-life (3–10 min), a brief offset of action, and immediate recovery of the clinical effect after interruption of the administration. It is metabolized by blood and tissue non-specific esterases, regardless of renal and hepatic metabolisms. It has a low volume of distribution and its plasma clearance rate is high (4). Non-specific esterase activity is present in preterm infants, irrespective of the gestational age (5). The usual adverse effects of remifentanil are similar to those observed with opioids, in particular bradycardia, hypotension, chest wall rigidity, nausea, and vomiting (6).

Several studies have evaluated its use for intubation of neonates, showing its feasibility (7–10). In 2016, we decided to change the intubation protocol at our NICU, with the introduction of remifentanil due to its specific properties, namely rapid onset of action, replacing our usual sedation protocol (sufentanil and atropine). This retrospective study evaluated the efficacy and safety of remifentanil as a premedication in preterm and full-term neonates undergoing elective endotracheal intubation in the first 18 months of use of this new protocol at our NICU.

## Materials and Methods

We conducted a retrospective study at the NICU of Port-Royal located in a university tertiary perinatal center at Cochin Hospital in Paris (France), from June 2016 to November 2017. The National Data Protection Authority (Commission Nationale de l'Informatique et des Libertés, CNIL n°1747084) approved this study. Under French regulations, this study is exempt from IRB review because it is an observational retrospective study using anonymized data from medical records.

All of the infants admitted to the NICU, regardless of age, weight, and whether they were full-term, who received remifentanil prior to an elective endotracheal intubation were included for any reason for intubation. A written protocol, accessible by all of the physicians, provided information regarding the remifentanil preparation and administration, as well as the cardiorespiratory monitoring during the procedure.

At our NICU, intubation is usually first performed by a pediatrician resident. If the intubation is unsuccessful after one or two attempts, the procedure is then performed by the attending neonatologist. The attempt is interrupted if the heart rate (HR) or the oxygen saturation (SpO2) become too low, at the discretion of the attending neonatologist.

### Dilution

The dilution was as follows: 1 mg remifentanil was diluted into 5 ml water or 5% glucose solution; 1 ml (equating to 200 μg of drug) of this dilution was added to 19 ml water or 5% glucose solution (1 ml = 10 μg of drug).

During the study period, the protocol for the administration of remifentanil was modified twice due to the occurrence of adverse events following the administration of remifentanil. The team hypothesized that serious side effects could be related to an excessive dose and therefore decided to reduce the total dose received. For the data analysis, we therefore distinguished three periods based on observed protocol changes.

### Study Periods

The first period (study period 1, SP1) was conducted from June to August 2016. Remifentanil was delivered at a dose of 0.5 μg/kg/min. The remifentanil infusion was stopped after successful intubation, without a maximum time limit. The second period (study period 2, SP2) was conducted from August to October 2016. The initial remifentanil dose was 0.25 μg/kg/min. If the sedation was insufficient, this dose could first be increased to 0.38 and then to 0.5 μg/kg/min if the previous dose was not effective. The remifentanil infusion was stopped after successful intubation, without a maximum time limit. During the third period (study period 3, SP3) from June to November 2017, the initial dose was 0.1 μg/kg/min. When the sedation was inadequate, this dose could be increased by 0.1 μg/kg every minute, with a maximum dose of 0.4 μg/kg/min. The infusion could not be extended beyond 10 min, with a maximum cumulative dose of 1 μg/kg. The remifentanil infusion was stopped when the laryngoscopy was started.

In the event of chest wall rigidity, the administration of a curare agent (mivacurium, 0.2 mg/kg) or an opioid antagonist (naloxone, 0.1 mg/kg) was an option.

It should be noted that the use of remifentanil was discontinued between SP2 and SP3 due to the occurrence of serious adverse reactions. During this period, the usual premedication protocol was used, namely sufentanil and atropine.

### Procedure

According to our local protocol, all infants routinely have cardiorespiratory, oxygen saturation, and non-invasive blood pressure monitoring during the intubation procedure. Firstly, Neopuff^TM^ (Fisher & Paykel, Healthcare, Auckland, New Zealand) mask ventilation is performed during the premedication time before laryngoscopy, with the parameters at the discretion of the intubator to achieve peripheral oxygen saturation >95%. Secondly, atropine (10 μg/kg) is administered intravenously as a bolus. Thirdly, a 0.5 mL bolus of remifentanil is administered to purge the catheter. Finally, continuous infusion of remifentanil is started. Laryngoscopy is performed when the patient appears to be sufficiently sedated according to the operator. The drugs are always administered by the proximal access of both peripheral intravenous or central venous catheters. At our NICU, physiological parameters (including the HR, the mean blood pressure (MBP), the SpO2, and the respiratory rate) and the duration of different attempts of intubation are recorded in the medical charts by a specific person every minute just before, during, and up to 10 min after the end of the laryngoscopy. Newborn pain is also assessed by a nurse before, during, and after intubation using the Acute Pain in Newborn Infants (APN) scale (11). As we recently changed the sedation protocol at our NICU, the operator had to report the quality of the intubation conditions on the medical charts according to the following scale adapted from Hans and Cooper (12–14):

- Excellent: Relaxed jaw, open vocal cords, no movement during endotracheal tube insertion
- Good: Relaxed jaw, open vocal cords, mild movements during endotracheal tube insertion.
- Acceptable: Mild jaw contraction and/or moving vocal cords and/or cough during endotracheal tube insertion.
- Poor: Jaw contraction or closed vocal cords or intense cough or rigidity during endotracheal tube insertion.

### Study Outcomes

The aim of our study was to determine the efficacy and the safety of continuous infusion of remifentanil for endotracheal intubation in neonates. The primary outcome was the quality of the intubation conditions under remifentanil as defined above. The secondary outcomes and process measurements included the number of intubation attempts, description of the procedure (including the duration of the remifentanil administration, the cumulative dose of remifentanil, and the total laryngoscopic time), the incidence of side effects, and variation of the physiological parameters. Bradycardia, hypotension, and chest wall rigidity are known side effects of remifentanil (6). Severe bradycardia, hypotension, and desaturation after remifentanil administration were defined as a decrease in the HR, the MBP, and an SpO2 higher than 50% of baseline, respectively. We defined a difficult intubation as one that required three or more intubation attempts. Other respiratory problems were defined as a prolonged desaturation requiring a transient increase of the ventilatory settings to an unexpected level, excluding chest wall rigidity.

### Statistical Analysis

The data were expressed as means with the standard deviation if the distribution was normal and medians with the interquartile (IQ) range if the distribution was non-normal. For the continuous variables, the groups were compared using the Student's *t*-test for the parametric variables and the Mann-Whitney test for the non-parametric variables. The categorical variables were compared using Fisher's exact test. The statistical analysis was performed using Stata 13.0 software (Stata Corporation, College Station, TX, USA), and a *p*-value of <0.05 was considered as statistically significant.

## Results

### Patient Characteristics

From June to October 2016 (SP1 and SP2) and from June to November 2017 (SP3), a total of 54 neonates with a birth gestational age of 23^+6^ weeks of gestation (WG) to 41^+3^ WG (median 26.7 WG, IQ 25-75: 25.3-29) and a birth weight from 590 g to 3,990 g (median 828 g, IQ 25-75: 705-1,105 g) were included. Sixteen patients were intubated during SP1, 10 during SP2, and 28 during SP3. At the time of the intubation, the patients were 0 to 78 days old (median 6.5 days, IQ 25-75: 2-17), and weighed between 530 and 3,990 g (median 908 g, IQ 25-75: 735-1,240 g). The baseline characteristics of the patients were similar during SP1, SP2, and SP3 (Table 1).

**Table 1:** The baseline characteristics of the patients.

**Characteristics**				**Total procedures (*N =* 54)**	**Comparison**
	**SP1 (*N =* 16)**	**SP2 (*N =* 10)**	**SP3 (*N =* 28)**	**SP1 + SP2 + SP3**	**SP1 + SP2/SP3 (*p*)**
Gestational age at birth (weeks), median (IQ 25-75)	27.3 (25.5–29)	29.2 (27.3–29.3)	26.1 (25–27.6)	26.7 (25.3–29)	0.07
Birth weight (g), median (IQ 25–75)	828 (673–1,018)	823 (750–1,125)	818 (725–1,120)	828 (705–1,105)	0.60
Female, *n* (%)	6 (38)	5 (50)	15 (54)	26 (48)	0.43
Postmenstrual age at intubation (weeks), median (IQ 25–75)	30.0 (28.2–31.5)	29.3 (27.6–31.9)	27.5 (26.4–31.4)	28.4 (26.6–31.9)	0.11
Age at intubation (day), median (IQ 25–75)	11 (5–20)	6 (1–12)	4.5 (1–13.5)	6.5 (2–17)	0.27
Weight at intubation (g), median (IQ 25–75)	988 (743–1,195)	880 (700–1,330)	898 (743–1,593)	908 (735–1,240)	0.86
Baseline vital signs, median (IQ 25–75)					
Heart rate (beats per minute)	166 (150–174)	166 (155–187)	159 (149–174)	163 (150–175)	0.31
Mean blood pressure (mmHg)	59 (47–64)	51 (42–60)	54 (48–62)	55 (46–62)	0.60
Oxygen saturation (%)	99 (95–100)	100	100 (96–100)	100 (96–100)	0.72
Respiratory rate (per minute)	43 (33–62)	(98–100) 48 (38–52)	41 (38–46)	41 (37–48)	0.53
Reason for intubation, *n* (%)					0.26
Respiratory failure	13 (81)	6 (60)	21 (75)	40 (74)	
Necrotizing enterocolitis	3 (19)	3 (30)	2 (7)	8 (15)	
HIE	0 (0)	1 (10)	3 (11)	4 (7)	
Surgery	0 (0)	0 (0)	1 (4)	1 (2)	
Magnetic resonance imaging	0 (0)	0 (0)	1 (4)	1 (2)	
Intubator, *n* (%)					0.76
Neonatologist in attendance	13 (81)	7 (70)	19 (70)	39 (74)	
Pediatric resident	3 (19)	3 (30)	8 (30)	14 (26)	
Intravenous access, *n* (%)					0.49
Percutaneous intravenous central catheter	6 (38)	4 (40)	9 (32)	19 (35)	
Umbilical venous catheter	4 (25)	3 (30)	12 (43)	19 (35)	
Peripheral venous access	6 (38)	3 (30)	7 (25)	16 (30)	
Ventilation before intubation, *n* (%)					0.67
Spontaneous ventilation	1 (6)	1 (10)	4 (14)	6 (11)	
Non–invasive ventilation	14 (88)	9 (90)	24 (86)	47 (87)	
Invasive ventilation	1 (6)	0 (0)	0 (0)	1 (2)	

*IQ, interquartile. HIE, Hypoxic–ischemic encephalopathy*.

### Procedure

The most frequent indication for elective intubation was respiratory failure (*n* = 40, 74%). There were 8 intubations for necrotizing enterocolitis (15%), 4 for hypoxic-ischemic encephalopathy (7%), 1 for surgery (2%), and 1 for MRI (2%) (Table 1). Eighteen patients (33%) were intubated at the first attempt, 23 (43%) at the second, 12 (22%) at the third, and 1 (2%) at the fourth. For 86% of the failed intubations (31/36), the first intubator was a resident. The median laryngoscopy time was 4 min (IQ 25-25: 2-8). The median remifentanil infusion duration was 4 min (IQ 25-75: 3-8) and the median cumulative dose was 1 μg/kg (IQ 25-75: 0.6-3) (Table 2). Thirteen neonates received a cumulative dose of more than 3 μg /kg.

**Table 2:** Outcomes of interest.

**Parameters**	**SP1 (*N =* 16)**	**SP2 (*N =* 10)**	**SP3 (*N =* 28)**	**Total procedures (*N =* 54) SP1 + SP2 + SP3**	**Comparison SP1 + SP2/SP3 (*p*)**
Duration of remifentanil administration (minutes), median (IQ 25–75)	6 (2–12)	8 (7–11)	4 (2–4)	4 (3–8)	<0.001
Cumulative dose of remifentanil (μg/kg), median (IQ 25–75)	3 (1–6)	3 (2–3.3)	0.6 (0.3–1)	1 (0.6–3)	<0.001
Total laryngoscopic time (minutes), median (IQ 25–75)	5 (2–8)	3 (1–4)	5 (2–8)	4 (2–8)	0.62
Attempts for successful intubation, *n* (%)					0.44
First attempt	4 (25)	5 (50)	9 (32)	18 (33)	
Second attempt	9 (56)	4 (40)	10 (36)	23 (43)	
Third attempt	3 (19)	1 (10)	8 (29)	12 (22)	
Fourth attempt	0 (0)	0 (0)	1 (4)	1 (2)	
Change in heart rate > 50 % of baseline during procedure, *n* (%)	5 (31)	2 (20)	5 (18)	12 (23)	0.51
Change in oxygen saturation > 50% of baseline during procedure, *n* (%)	6 (38)	5 (50)	9 (32)	20 (37)	0.41
Change in mean blood pressure > 50% of baseline procedure, *n* (%)	0 (0)	0 (0)	2 (7)	2 (4)	0.49
Change in respiratory rate > 50% of baseline procedure, *n* (%)	2 (13)	2 (20)	2 (7)	6 (11)	0.42
Additional Epinephrine given, *n* (%)	1 (6)	1 (10)	0 (0)	2 (4)	0.23
Additional Naloxone given, *n* (%)	0 (0)	0 (0)	0 (0)	0 (0)	–
Additional Mivacurium given, *n* (%)	0 (0)	0 (0)	1 (4)	1 (2)	–
APN score (0–10)^a^, median (IQ 25–75)					
Before intubation	2 (0–7)	1 (0–1)	5 (2–7)	2 (0–5)	0.09
During intubation	0 (0–2)	0 (0–1)	1 (0–3)	1 (0–2)	0.13
After intubation	0 (0–1)	0 (0–0)	0 (0–1)	0 (0–0)	0.52
Adverse events, *n* (%)					
Chest wall rigidity	3 (19)	0 (0)	3 (11)	6 (11)	1.0
Other respiratory problems^b^	3 (19)	2 (20)	0 (0)	5 (9)	0.02
Laryngospasm	0 (0)	1 (10)	0 (0)	1 (2)	0.48
Total number of adverse events^c^	6 (38)	3 (30)	3 (11)	12 (22)	0.05

*IQ: interquartile. SD: standard deviation*.

a *APN: Acute Pain in Newborn infants score of 0 (no pain) to 10 (highest pain) comprising 3 assessments (facial responses, limb movements, and vocal expression of pain). The APN scores were available for 23 intubations*.

b *Other respiratory problems were defined as a prolonged desaturation requiring a transient increase of ventilatory settings at an unexpected level, excluding chest wall rigidity*.

c *Number of adverse events per total number of procedures*.

### Efficacy of Remifentanil as a Sedative

The intubating conditions, as assessed by the intubator, were excellent for 37 procedures (69%), good for 10 procedures (18%), acceptable for 5 procedures (9%), and poor for 2 procedures (4%) (Table 3). The APN scores were only available for 23 intubations (43%). The median APN score was 2 (IQ 25-75: 0-5) before the sedation, 1 (IQ 25-75: 0-2) during the laryngoscopy, and 0 (IQ 25-75: 0-0) after the intubation (Table 2).

**Table 3:** Assessment of the intubating conditions.

**Intubating conditions**				**Total procedures (*N =* 54)**	**Comparison**
	**SP1 (*N =* 16)**	**SP2 (*N =* 10)**	**SP3 (*N =* 28)**	**SP1 + SP2 + SP3**	**SP1 + SP2 /SP3 (*p*)**
Assessment of sedation, *n* (%)					
Poor	0	0	2 (7)	2 (4)	0.71
Acceptable	1 (7)	1 (10)	3 (11)	5 (9)	
Good	3 (19)	2 (20)	5 (18)	10 (18)	
Excellent	13 (87)	7 (70)	17 (61)	37 (69)	

### Variation of the Physiological Parameters

Twelve patients (23%) had a decrease in the HR of more than 50% of baseline during the procedure and 20 (37%) had a decrease in the SpO2 of more than 50% of baseline (Table 2).

### Complications

The main side effect associated with remifentanil administration was chest wall rigidity, observed in 6 procedures (11%), requiring administration of epinephrine in 2 procedures, which in one case was associated with chest compressions. No procedure required the administration of mivacurium or naloxone. Five patients (9%) were affected by other complications, one of whom required chest compressions. Laryngospasm occurred on one occasion, requiring advanced resuscitation and epinephrine. Five of the 13 patients who received more than 3 μg/kg of remifentanil had side effects (1 chest wall rigidity, 1 laryngospasm, and 3 other respiratory problems; Table 4).

**Table 4:** Characteristics of the procedures with adverse events.

**Patient**	**Adverse event**	**Study period**	**At intubation**			**Intubator**	**Intravenous access**	**Duration of remifentanil administration (minutes)**	**Cumulative dose of remifentanil (μg/kg)**	**Total laryngoscopic time (second)**	**Number of attempts**
			Age^*^ (day)	Post–menstrual age^*^ (weeks)	**Weight (g)**						
1	Respiratory problems	1	0–5	33	1,775	A	UVC	5	2.5	2	2
2	Chest rigidity	1	21–25	30	1,235	A	PVC	2	1	1	2
3	Respiratory problems	1	41–45	32	1,125	R	PVC	N/A	N/A	N/A	1
4	Chest rigidity	1	6–10	26	735	A	PCVC	12	6	3	2
5	Chest rigidity	1	21–25	29	885	A	PVC	1	0.5	30	3
6	Respiratory problems	1	0–5	25	750	A	UVC	10	5	7	2
7	Respiratory problems	2	26–30	33	1,570	A	PVC	10	3.67	3	2
8	Respiratory problems	2	6–10	30	1,030	R	PCVC	11	4.05	1	1
9	Laryngo–spasm	2	0–5	28	820	A	UVC	12	3	1	2
10	Chest rigidity	3	31–35	32	1,800	A	PVC	N/A	N/A	27	2
11	Chest rigidity	3	11–15	27	915	R	PCVC	3	0.6	24	3
12	Chest rigidity	3	0–5	38	3,160	A	UVC	4	1	6	2

*A, neonatologist in attendance; R, pediatric resident; UVC, umbilical venous catheter; PVC, peripheral venous catheter; PCVC, percutaneous central venous catheter; N/A, not available*.

* *At intubation, age is presented as a 5–day range and postmenstrual age as a 1–week range to avoid identifying patient data*.

### Comparisons Between SP1 and SP2, and SP3

The cumulative dose of remifentanil was not significantly different between SP1 and SP2, whereas it was significantly lower for SP3. To determine if there was a dose-dependent effect of remifentanil on the occurrence of side effects, we decided to compare SP1-SP2 to SP3. The median remifentanil infusion duration and the median cumulative dose were significantly lower during SP3 than SP1-SP2 (*p* < 0.001). There were 3 occurrences of chest wall rigidity (11%) during SP1-SP2 and 3 (11%) during SP3 (*p* = 1.0). The single laryngospasm occurred during SP1-SP2. All of the other respiratory problems were observed during SP1-SP2 (*n* = 5, 19%, *p* = 0.02). The total number of adverse events was more frequent during SP1-SP2 than during SP3 [*n* = 9 (35%), vs. *n* = 3 (11%), *p* = 0.05] (Tables 2, 3).

## Discussion

In this retrospective study, we observed that continuous administration of remifentanil after an atropine bolus led to excellent or good intubating conditions for 87% of the neonates, although side effects—including chest wall rigidity (11%), other respiratory problems (9%), and laryngospasm (2%)—occurred in 22% of the procedures. In addition, 23% of the procedures were complicated by severe bradycardia and 37% by severe desaturation. Given the good intubating conditions and the high incidence of adverse effects, the exclusive use of high doses of remifentanil and the merits of premedication before intubation may warrant critical evaluation. Indeed, it seems inappropriate to consider reaching the level of general anesthesia with an opioid alone. When combined with midazolam or propofol, 1 μg/kg of remifentanil appears to be sufficient to obtain quality sedation without side effects (8, 15, 16). Therefore, one of our hypotheses is that the doses were too high because the physician wanted to achieve general anesthesia with remifentanil alone.

Chest wall rigidity was the main severe side effect associated with remifentanil administration, as also observed in other studies. For example, a randomized double-blind trial performed by Choong et al. compared preterm and full-term newborns who received remifentanil (a bolus of 3 μg/kg over 60 s) and normal saline placebo vs. fentanyl and succinylcholine for intubation premedication (7). Two cases of chest wall rigidity (13.3%) were described in the remifentanil group and none in the fentanyl group. The lack of chest wall rigidity in the fentanyl group could, however, be attributed to the systematic use of succinylcholine. Another randomized trial compared preterm newborns who received remifentanil (a bo1us of 1 μg/kg over 60 s) vs. morphine and midazolam for premedication of intubation (17). In the remifentanil group, 2 patients had chest wall rigidity (5.5%) while there were none in the morphine group. In a prospective study, de Kort et al. described chest wall rigidity in 6 patients (43%), and they discontinued the study prematurely due to side effects and a lack of efficacy (18). Conversely, other studies have not shown chest wall rigidity with remifentanil (8–10). The absence of an objective scale to assess chest wall rigidity may partly explain the variability of its occurrence from one study to another, as its evaluation was subjective and operator-dependent.

The total received dose of remifentanil could be one of the factors that determine the occurrence of side effects such as chest wall rigidity. For endotracheal intubation, remifentanil as a single premedication is typically administered at doses ranging from 1 to 3 μg/kg over 60 s (8–10). In our study, side effects tended to be more common during SP1-SP2 than SP3 (35% vs. 11%, *p* = 0.05), and the median cumulative dose of remifentanil was significantly higher during SP1-SP2 than in SP3 (3.42 μg/kg vs. 0.66 μg/kg, *p* < 0.01). Moreover, 5 of the 13 patients who received more than 3 μg/kg of remifentanil had side effects (1 chest wall rigidity, 1 laryngospasm, and 3 other respiratory problems). As previously discussed for the de Kort study (19), a cumulative dose of remifentanil <3 μg/kg may partly promote chest wall rigidity. These data suggest that there may be a dose-dependent effect for the occurrence of adverse events associated with remifentanil. However, in our study, some newborns exhibited adverse events irrespective of the remifentanil dose (5 patients had side effects and received 0.5 to 1 μg/kg of remifentanil), which suggests interindividual variability.

The procedures with remifentanil were also associated with transient severe bradycardia, despite routine administration of a vagolytic agent, and severe desaturation, with a stable MBP. Severe bradycardia and desaturation could be explained in part by the use of remifentanil, but also by poor analgesia, an excessively long duration of laryngoscopy potentially related to the lack of experience of the intubator, and/or respiratory problems. We cannot draw conclusions in our retrospective study regarding the responsibility of remifentanil in the occurrence of these severe side effects, but they are reason for a high level of caution regarding its use.

To reduce the side effects of remifentanil, continuous administration was preferred to bolus injection, but the incidence of such events appeared to be similar to those observed in the literature with bolus administration (7, 9).

Remifentanil as a single premedication provided excellent and good intubating conditions in 87% of the procedures, even when a low dose was used. This finding is consistent with the study of Shin et al. who demonstrated that low doses of remifentanil (0.1 to 0.25 μg/kg/min) were effective in neonates (10). Despite these excellent and good intubating conditions, only 33% of the patients were successfully intubated on the first attempt, which could be seen to be inconsistent. This poor outcome could be explained in part by the fact that in 74% of the cases, the intubator was an attending neonatologist, although a lack of clinical experience should not be a cause of intubation failure.

Other drugs (i.e., ketamine or propofol) have been proposed for sedation and analgesia in neonates, but their use is still controversial. Propofol has been associated with bradycardia, desaturation, and prolonged hypotension in newborns, and it is not an analgesic agent (14, 20, 21). Further research is needed to establish safety profiles for the use of ketamine in neonates due to concerns regarding possible neurotoxicity in animal studies (22). Regarding remifentanil, studies performed in mice have shown that it has an antiapoptotic impact and that it exerts beneficial effects against excitotoxicity on the developing mouse brain that is associated with a reduction in the brain lesion size as well as prevention of a number of behavioral deficits in young mice (23, 24). It should be noted that the neurotoxicity of some anesthetic agents has mainly been demonstrated in animals, and most of the time in the absence of painful procedures. The potential neurotoxicity of the anesthetic could, therefore, be counterbalanced by the beneficial effect of pain reduction. In humans, a recent randomized trial (GAS Trial) did not reveal neurodevelopmental disorders at 5 years of age after exposure to <1 h of general anesthesia before 3 months of life (25).

This study has several limitations. First, due to the retrospective design of our study, some of the data were missing, including the APN evaluation, which was only available for 23 (45%) of the procedures. This high level of missing data for the APN score in our study does not allow us to draw any conclusions regarding the analgesic effect of remifentanil in case of intubation. A second limitation is the relatively small population (54 patients). In addition, the protocol was changed twice during the study period, further reducing the sample size for each subgroup. This led us to compare SP1-SP2 with SP3, and thus compare a group with high doses of remifentanil vs. a group with low doses, but the number of patients in each group was still limited (*n* = 26 for SP1-SP2 and *n* = 28 for SP3). Thirdly, we only used a vagolytic and an opioid agent before intubation. International guidelines, such as those of the American Academy of Pediatrics, recommend the use of an analgesic agent or an anesthetic dose of a hypnotic drug, a vagolytic, and a rapid-onset muscle relaxant before a nonemergent intubation in neonates. (2). In this study, a muscle relaxant was not used in order to evaluate the effect of remifentanil. Therefore, the use of a muscle relaxant should decrease the incidence of respiratory problems, in particular chest wall rigidity and should improve intubating conditions.

## Conclusion

In our study, the administration of remifentanil as premedication for intubation in preterm and full-term newborns provided adequate intubating conditions. Nevertheless, adverse effects (such as chest wall rigidity) are a potential risk, possibly related to the total dose received.

In our opinion, these data do not indicate that the use of remifentanil alone prior to intubation is safe, given the occurrence of side effects in 22% of the procedures. The use of remifentanil with a minimum effective dose in combination with curare and possibly a hypnotic agent could be safer and should be evaluated in future trials.

## Data Availability Statement

The datasets analyzed in this manuscript are not publicly available. Requests to access the datasets should be directed to Clement Chollat, clement.chollat@gmail.com.

## Ethics Statement

Ethical review and approval was not required for the study on human participants in accordance with the local legislation and institutional requirements. Written informed consent from the participants' legal guardian/next of kin was not required to participate in this study in accordance with the national legislation and the institutional requirements.

## Author Contributions

CC contributed to the design of the study, the analyses, the data collection, and the drafting of the manuscript. AM collected the data and drafted the manuscript. M-SA performed the statistical analysis and contributed to the drafting of the manuscript. CG contributed to the data collection and the analyses, and they revised the manuscript. JP contributed to the analyses of data, and they revised the manuscript. EZ-T contributed to the analyses of the data, and they revised the manuscript. AK contributed to the design of the study, the data collection, and they contributed significantly to the drafting of the manuscript. HT contributed to the analyses of the data, and they revised the manuscript. P-HJ contributed to the design of the work and they critically reviewed the manuscript for important intellectual content. All of the authors participated significantly in the review, revision, and approval of the version to be published, and they have agreed to be accountable for all aspects of the work in ensuring that questions related to the accuracy or integrity of any part of the work are appropriately investigated and resolved.

### Conflict of Interest

The authors declare that the research was conducted in the absence of any commercial or financial relationships that could be construed as a potential conflict of interest.
